# Exploring novel heterojunctions based on the cerium metal–organic framework family and CAU-1, as dissimilar structures, for the sake of photocatalytic activity enhancement†

**DOI:** 10.1039/d2ra06034e

**Published:** 2022-11-10

**Authors:** Moein Darabi Goudarzi, Negin Khosroshahi, Vahid Safarifard

**Affiliations:** Department of Chemistry, Iran University of Science and Technology Tehran 16846-13114 Iran vsafarifard@iust.ac.ir

## Abstract

Ce-based metal–organic frameworks (Ce-MOFs) are excellent photocatalysts due to their high efficiency in charge transportation. The integration of this family with CAU-1 (CAU standing for Christian-Albrechts-University), as a MOF benefiting from its ultra-high surface area, can remarkably enhance the properties of the structure. This research includes four new heterojunctions, namely CAU-1/Ce-BDC–NH_2_, CAU-1/Ce-UiO-66, CAU-1/Ce-MOF-808, and CAU-1/Ce-BDC, prepared by an innovative method, and several characterization techniques were employed to study the structural features of the frameworks. Their high surface area and low bandgap energy seemed appropriate for catalytic applications. Therefore, CAU-1/Ce-BDC was chosen for the photocatalytic removal of Cr(vi), a dangerous heavy metal, from aqueous systems. According to the results, a 96% reduction of Cr(vi) to Cr(iii) within 75 min was observed, and the catalyst retained its stability after four runs of reactions under acidic conditions.

## Abstract 2

Ce-based metal–organic frameworks (Ce-MOFs) are excellent photocatalysts due to their high efficiency in charge transportation.

## Graphical abstract

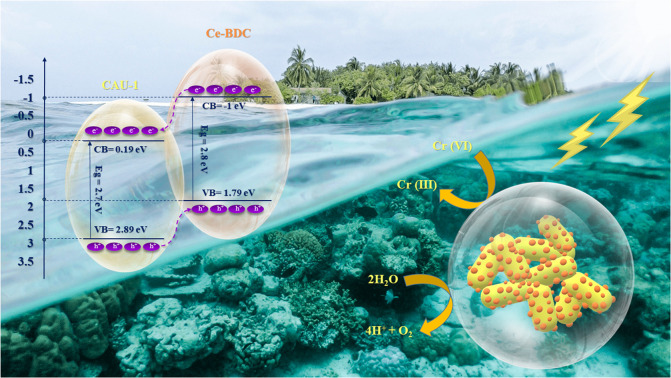

## Introduction

Environmental issues resulting from excessive consumption of fossil fuels have attracted the world's attention toward finding a strategy to replace renewable and green energy with familiar energy sources.^1^ Converting solar energy *via* photothermal, photovoltaic, and photocatalytic approaches can lead us to a cleaner planet.^2^ For instance, in photocatalytic reactions, the photocatalyst absorbs sunlight, and the wavelengths that fit its bandgap cause electron excitation to the conductive band. The charge separation starts photocatalytic reactions.^3–5^

Countries are obsessed with discharging pollutants into wastewater, especially heavy metals such as cadmium, chromium, lead, copper, nickel, and thallium.^6^ Chromium (vi) is one of the most dangerous heavy metals, very small amounts of which, even at parts per billion levels, can be highly toxic.^7^ Dichromate (Cr_2_O_7_^2−^), chromate (CrO_4_^2−^), and hydrogen chromate (HCrO_4_^−^) are three primary Cr(vi) compounds that are highly soluble in water.^8–10^ Therefore, chromium is considered as a Group “A” carcinogen by the United States Environmental Protection Agency (EPA) because of its severe effects on human health.^11–13^ However, unfortunately, seepage or careless disposal while working with chromium in various industries including photography, leather tanning/dyeing, mining, electroplating, metallurgy, and smelting over the years has caused environmental issues.^14,15^ Owing to the co-existence of Cr(vi) with other ions and its high toxicity in water, introducing an efficient and cost-effective solution for reducing this ion to Cr(iii), which is less toxic at low concentrations, has attracted scientists' attention.^16^ Chemical reduction, biological reduction, electrochemical reduction, and photocatalytic reduction are the most common reduction methods available to overcome this challenge.^10,17,18^ Numerous materials, including semiconductors (TiO_2_, ZnO, Fe_2_O_3_, *etc.*) and metal–organic frameworks (MOF-808, UiO-66, MIL-125, *etc.*) can perform photocatalytic reactions perfectly.^19–21^

The conjugation of transition metals with organic linkers results in metal–organic frameworks (MOFs).^22^ These porous structures have advantages of high specific surface area, adjustable pore volume, and structure flexibility,^23^ which are critical factors in gas storage/and separation,^24^ sensing,^25^ photocatalysis,^26^ and drug delivery.^27^ Due to these features, more than 100 000 MOFs have been synthesized so far.^28^ Combining MOFs with other materials including silica, noble metal particles, polystyrene, and magnetic particles is an excellent strategy to produce composites with better efficiency in the selected application.^29–31^ However, it is noteworthy that obtaining a hybrid MOF or a MOF-on-MOF structure by synthesizing a MOF in the presence of another MOF is a graceful method for fabricating heterojunctions that can enhance the structural properties.^32,33^ The epitaxial growing method (EGM) and the surfactant assistance method are well-known strategies in this field.^34^ The main feature of these methods is the growth of one MOF on the surface of another MOF.^30,35,36^ Core–shell, asymmetric, yolk–shell, core-satellite, hollow multi-shell, and film on film are typical MOF-on-MOF morphologies.^37^ Notably, knowing the components and structures of the participating MOFs is vital for successfully synthesizing MOF-on-MOF structures. Fe-MIL-88B@Ga-MIL-88B,^38^ ZIF-8@ZIF-67,^39^ and Zr-MOF-808@Ce-MOF-808 are some examples reported by scientists in previous papers. However, growing a MOF on another MOF with a different structure and component is quite a challenge;^40^ therefore, the number of this kind of heterojunction is smaller than the above-mentioned types with similar structures or components.

The research on novel efficient photocatalysts focused our attention on cerium-based MOFs. In the current project, we report an advanced approach to obtain CAU-1/Ce-BDC–NH_2_, CAU-1/Ce-UiO-66, CAU-1/Ce-MOF-808, and CAU-1/Ce-BDC as four new MOF-on-MOF heterojunctions, both the structures and components of which are different in each MOF-on-MOF structure. A cerium-based MOF family, which is known as a stable group of MOFs that display excellent photocatalytic performance, was successfully grown on the surface of CAU-1, an Al-based MOF, and it was validated by various characterization techniques. Moreover, the comparison of each nanocomposite with its components and other synthesized MOF hybrids' efficiency in Cr(vi) was explored as a proof of concept. CAU-1, as the interior part, possesses an absorbent. In contrast, the exterior part is responsible for communicating with the surrounding media, so the nanocomposite's dispersity and stability depend on this part. As a result of the combination of the two structures, the synergetic effect of MOF-on-MOF structures has been improved, and therefore, the new constructions demonstrate superior photocatalytic activity compared with bare MOFs.

## Experimental section

### Chemicals

All the reagents were of analytical grade and used without further purification. Cerium(iii) chloride heptahydrate (CeCl_3_·7H_2_O), aluminum chloride (AlCl_3_), CAN: cerium(iv) ammonium nitrate ((NH_4_)_2_Ce(NO_3_)_6_, Sigma-Aldrich), 1,3,5-benzenetricarboxylic acid (BTC, 98%), 1,4-benzenedicarboxylic acid (H_2_BDC), 2-aminoterephthalic acid (NH_2_BDC), sodium sulfate (Na_2_SO_4_), carbon black, *N*-methyl-2-pyrrolidone (NMP), polyvinylidene fluoride (PVDF), formic acid (CH₂O₂), *N*,*N*-dimethylformamide (DMF, C_3_H_7_NO, 99.5%), methanol (CH_3_OH, 99.7%), acetone and other necessary chemicals were procured from Merck company and used without further purification.

### Synthesis methods

#### Preparation of CAU-1

CAU-1 ([Al_4_(OH)_2_(OCH_3_)_4_(NH_2_BDC)_3_]) was prepared according to a previously reported procedure with slight modification.^41^ Briefly, AlCl_3_ (12.3 mmol, 2.967 g) and BDC–NH_2_ (4.1 mmol, 0.746 g) were mixed in a beaker. Then, 30 mL of methanol was slowly added to the mixture. The solution was sonicated for 10 min after cooling down. Afterward, the mixture was transferred into a 100 mL autoclave and heated to 125 °C for 5 h. The yellow CAU-1 powder was obtained after centrifugation and washing three times with methanol. Finally, it was dried in a vacuum oven at 100 °C for 12 h (CCDC code: 723320).

#### Preparation of CAU-1/Ce-BDC–NH_2_

**Fig. 1:**
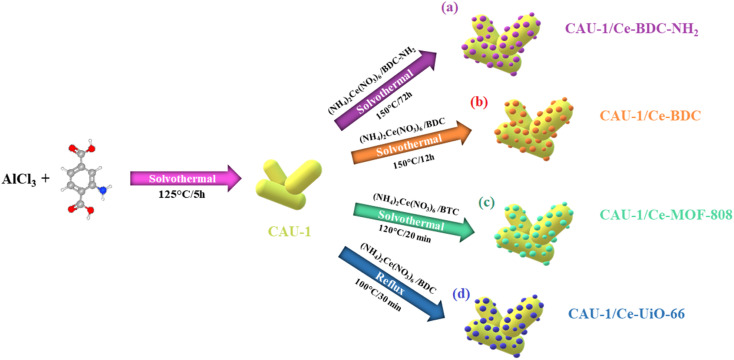
Synthesis process of (a) CAU-1/Ce-BDC–NH_2_, (b) CAU-1/Ce-BDC, (c) CAU-1/Ce-MOF-808, and (d) CAU-1/Ce-UiO-66 MOF-on-MOF heterostructures.

CAU-1 (0.22 g), BDC–NH_2_ (1.086 g), and CAN (822.34 mg) were solved in DMF (10 mL), DMF (18 mL), and methanol (2 mL) in three beakers, respectively. After complete dispersion, all solutions were mixed and stirred for 30 min. Then, the resulting liquid was moved to a high-pressure Teflon-lined stainless-steel autoclave and heated at 150 °C for 72 h. After washing and centrifuging the precipitate, a light brown powder was obtained, and it was also kept in a vacuum oven at 100 °C for 12 h to activate the structure (Fig. 1a).

#### Preparation of CAU-1/Ce-BDC

*Via* a solvothermal route, in a beaker, CAN (1 g) and H_2_BDC (0.6 g) were suspended in DMF (10 mL). In another beaker, CAU-1 (0.253 g) was dissolved in DMF (10 mL) and added to the first beaker. After complete dispersion, the mixture was moved to a Teflon-lined stainless-steel autoclave and heated at 150 °C for 12 h. After washing and centrifuging with DMF three times, an ivory powder was obtained and activated in a vacuum oven at 100 °C for 12 h (Fig. 1b).

#### Preparation of CAU-1/Ce-MOF-808

CAU-1 (0.151 g), H_3_BTC (0.224 g), and CAN (1.753 g) were solved in DMF (10 mL), DMF (12 mL), and distilled water (6 mL) in three separate beakers, respectively. Each beaker was let to be dispersed entirely under sonication for 20 min. The contents of the first two beakers were mixed and sonicated for 10 min. Next, the contents of the third beaker were added to the solution, as mentioned above. After adding 2.57 mL of formic acid, it was transferred into a vial and placed in an oven at 120 °C for 20 min. The light brown powder was washed and centrifuged with DMF and acetone several times and kept in a vacuum oven at 100 °C for 12 h (Fig. 1c).

#### Preparation of CAU-1/Ce-UiO-66

In a beaker, H_2_BDC (0.531 g) was dissolved in DMF (12 mL) and poured into another beaker containing a mixture of CAU-1 (0.123 g) dispersed in DMF (10 mL). After 15 min of sonication, CAN (1.753 g) dispersed in distilled water (6 mL) in the third beaker was added to the aforementioned solution. After extra sonication, a light brown precipitate was obtained by placing the mixture in a preheated oil bath at 100 °C for 15 min. It was washed and centrifuged several times with DMF and acetone, followed by activation in a vacuum oven at 100 °C for 12 h (Fig. 1d).

### Material characterization

Infrared spectra were recorded using a Nicolet 100 Fourier Transform IR spectrometer in the range of 500–4000 cm^−1^ with a KBr disk. X-ray powder diffraction (XRD) measurements were performed using a Philips X'pert diffractometer with monochromatic Cu-Kα radiation (*λ* = 1.54056 Å). The Mercury software prepared the simulated XRD powder pattern based on single-crystal data. The sonicator used in this study was a Samkoon Sonicator with adjustable power output (maximum 400 W at 20 kHz). The elemental composition of the nanocatalyst was studied by EDX analysis using a TESCAN4992. The samples were characterized using a scanning electron microscope (SEM) (Philips XL 30 and S-4160) with gold coating. Transmission electron microscopic (TEM) images were provided using a Zeiss EM 900 electron microscope (Germany) operating at 80 kV. UV-visible DRS spectra were recorded using a Shimadzu MPC-2200 spectrophotometer. Fluorescence emission was performed using a Cary Eclipse MY13250011 (*λ*_exc_ = 245 nm). The electrochemical measurements were carried out using a Metrohm Autolab PGSTAT204.

### Photocatalytic Cr(vi) reduction

In a common Cr(vi) reduction process, following the preparation of Cr(vi) solution (50 mL, 10 ppm), specific amounts of catalyst, hydrochloric acid, and benzyl alcohol, as the hole scavenger, were added to reach the adsorption–desorption equilibrium under continuous magnetic stirring at room temperature for 30 min in the darkness. After visible light irradiation, the change in the color of the mixture was a sign of reaction progression. Then, the catalyst was separated *via* centrifugation, and the amount of chromium in the supernatant solution was measured using a UV-vis spectrophotometer at 350 nm. Different parameters including catalyst dosage, chromium concentration, water source, and pH were tested, and the results were compared to obtain the optimum condition. What is more, the following relation was used to determine the photocatalytic efficiency:(*C*_ο_ − *C*_t_)/*C*_ο_ × 100where *C*_ο_ and *C*_t_ represent the initial concentration of Cr(vi) species in mg L^−1^ and the concentration at the reaction time in mg L^−1^, respectively.

### Photoelectrochemical measurements

An electrochemical Metrohm Autolab PGSTAT204 was employed to investigate the frameworks' photoelectrochemical properties. The counter, reference, and working electrodes were used in a three-electrode system. A Pt wire served as the counter electrode and an Ag/AgCl electrode (in saturated KCl) as the reference electrode. Specifically, the working electrode was made by dispersing the as-prepared materials (75%) in a solution consisting of NMP, PVDF (5%), and carbon black (20%). To measure the electrochemical impedance spectra (EIS) and Mott–Schottky (MS) plots in an aqueous solution of Na_2_SO_4_ (0.2 M) as the electrolyte, the above-mentioned mixture was sprayed uniformly on the surface of a Ni foam and dried at 100 °C for 12 h for solvent removal.

## Results and discussion

### Structure and morphology

**Fig. 2:**
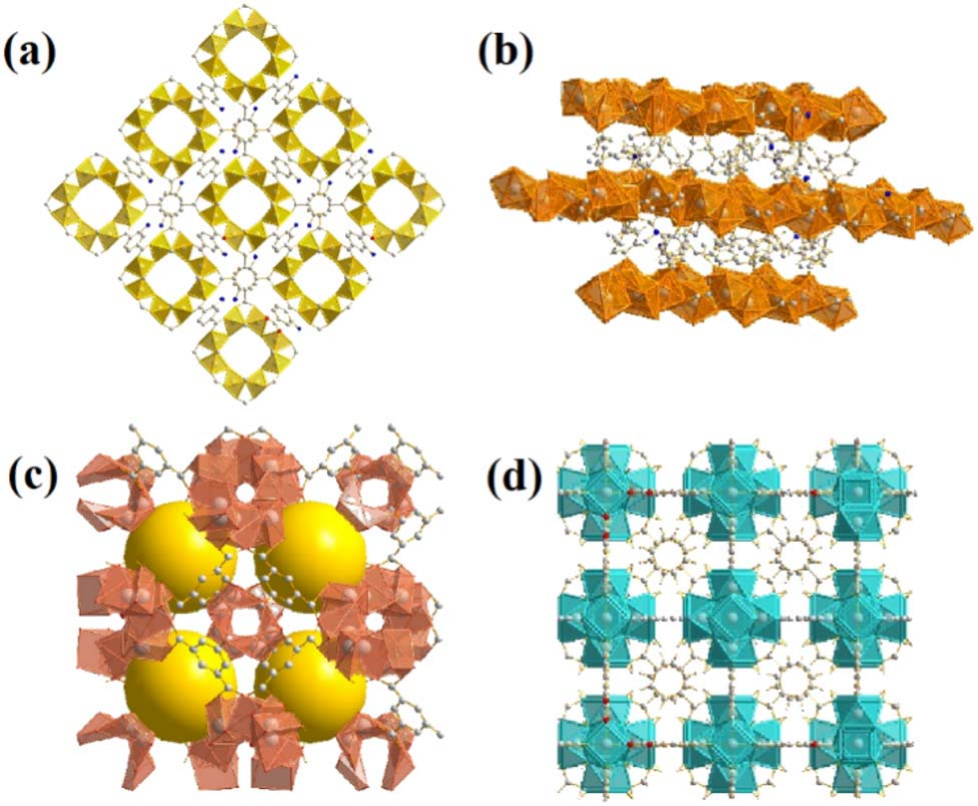
Images of as-prepared (a) CAU-1, (b) Ce-BDC–NH_2_ and Ce-BDC, (c) Ce-MOF-808, and (d) Ce-UiO-66.

From the four structures based on cerium, Ce-BDC–NH_2_ ([Ce_6_(NH_2_BDC)_9_(DMF)_6_(H_2_O)_3_]·3DMF) and Ce-BDC ([Ce_6_(BDC)_9_(DMF)_6_(H_2_O)_3_]·3DMF) as metal–organic frameworks based on Ce^3+^, Ce-MOF-808 ([Ce_6_(μ_3_-O)_4_(μ_3_-OH)_4_(BTC)_2_(OH)_6_(H_2_O)_6_] and Ce-UiO-66 ([Ce_6_O_4_(OH)_4_(BDC)_6_]) as MOFs based on Ce^4+^ were chosen to be grown on the surface of CAU-1 ([Al_4_(OH)_2_(OCH_3_)_4_(NH_2_BDC)_3_]), which is known for its ultra-high surface area (1816.3 m^2^ g^−1^) (Fig. S12†). A slight change in the oxidation number of cerium results in various frameworks. The main difference between frameworks synthesized *via* Ce^3+^ and Ce^4+^ is their topology (Fig. 2a–d).^42^ Contrary to isoreticular Ce-BDC–NH_2_ and Ce-BDC, which are two-dimensional frameworks, Ce-MOF-808 and Ce-UiO-66 are three-dimensional structures. Despite the presence of –NH_2_ groups on the linker of Ce-BDC–NH_2_, this structure could not exhibit high performance in photocatalytic Cr(vi) reduction reactions compared to Ce-BDC. This might be attributed to the steric hindrance of the BDC–NH_2_ linker. Additionally, Ce-BDC possesses more active sites and exhibits higher light absorption than Ce-MOF-808 and Ce-UiO-66, respectively. All these properties made Ce-BDC the optimal MOF and showed the highest efficiency in photocatalytic Cr(vi) reduction reactions, either alone or in combination with CAU-1 (Fig. 6b).

**Fig. 3:**
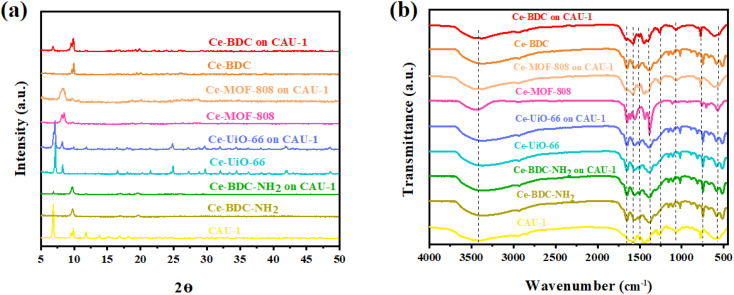
(a) Powder XRD pattern and (b) FT-IR spectra of CAU-1/Ce-BDC–NH_2_, CAU-1/Ce-BDC, CAU-1/Ce-MOF-808, and CAU-1/Ce-UiO-66 heterostructures and their related MOFs.

The X-ray diffraction information of all synthesized samples is provided in Fig. 3a. According to the patterns, it can be deduced that all CAU-1/Ce-BDC–NH_2_, CAU-1/Ce-UiO-66, CAU-1/Ce-MOF-808, and CAU-1/Ce-BDC are synthesized properly owing to the inclusion of characteristic peaks of both parts of each prepared heterojunction in their XRD pattern. CAU-1 existing in all composites displayed characteristic peaks at 2*θ* = 6.8°, 2*θ* = 9.9°, and 2*θ* = 13.8°, corresponding to the (011), (002), and (022) planes, respectively. The sharp and strong peak at 2*θ* = 6.8° was attributed to the methanol solution used in the synthesis procedure of CAU-1.^43^ Moreover, connecting the –OH functional group on the CAU-1 crystal contributes to the peak at 2*θ* = 9.9°.^41^ Ce-BDC–NH_2_ exposed three major peaks at 2*θ* = 9.7°, 2*θ* = 17.0°, and 2*θ* = 19.7°.^44^ Notably, it is reported that Ce-BDC–NH_2_ is isostructural with La-BDC–NH_2_.^45^ Five sharp reflections were observed in Ce-UiO-66 X-ray diffraction. 2*θ* = 7.1°, 8.2°, 11.7°, 13.7°, and 14.3° are the reflections that are referred to as the (111), (200), (220), (311), and (222) crystallographic planes, respectively.^46,47^ Ce-MOF-808 is another synthesized MOF that possessed a diffraction peak at 2*θ* = 8.14°, corresponding to the (311) plane, and another one at 2*θ* = 8.5°, that represents the (222) plane.^48,49^ The final MOF is Ce-BDC with two strong reflections at 2*θ* = 9.6° and 2*θ* = 9.9°, indicating high crystallinity like other mentioned MOFs.^50,51^

The presence of functional groups was analyzed by Fourier-transform infrared (FT-IR) spectroscopy to further comprehend the structure of Ce-MOFs and their synthesized heterojunctions. A similar peak was observed at exactly 3438 cm^−1^ for all samples. It is attributed to the O–H stretching vibration for Ce-MOF-808, Ce-BDC, Ce-UiO-66, and amino group for CAU-1 and Ce-BDC–NH_2_.^52–56^ The structures also showed symmetric and asymmetric stretching modes of carboxylic groups, and benzene ring bonding (C–H) in the range of 1400–1700 cm^−1^ and near 750 cm^−1^, respectively. Furthermore, the region of 500–700 cm^−1^ is the location of metal–O vibration in frameworks.^43,57–59^ Interestingly, all prepared heterojunctions displayed characteristic peaks of both CAU-1 and their secondary MOF as proof of the correctness of the synthesized structures (Fig. 3b).

**Fig. 4:**
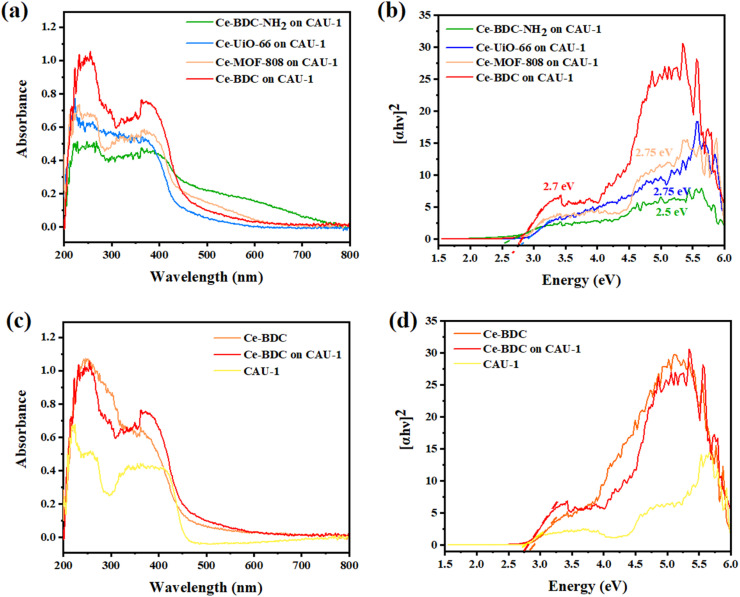
(a) UV-vis DRS spectra, (b) corresponding Tauc plots of (*αhν*)^2^*versus hν* of CAU-1/Ce-BDC–NH_2_, CAU-1/Ce-BDC, CAU-1/Ce-MOF-808, and CAU-1/Ce-UiO-66 heterojunctions, comprehensive, (c) UV-vis DRS spectra, and (d) corresponding Tauc plots of CAU-1/Ce-BDC with CAU-1 and Ce-BDC.

To measure the optical properties of structures and their photon absorption potential, UV-vis diffuse reflectance spectroscopy (DRS) was successfully undertaken in the range of 200–800 nm. Compared to pristine CAU-1, all synthesized heterojunctions exposed broader and more intense absorption peaks (Fig. 4a and c). The broad peak in the 200–450 nm region confirms their activity under visible light in addition to the UV spectrum.^60^ The absorption edge of Ce-BDC–NH_2_, Ce-UiO-66, Ce-MOF-808, Ce-BDC, and their related hybrid structures were obtained at around 260, 223, 230, and 255 nm, respectively. The sharp peak near 400 nm is also correlated with visible light utilization (Fig. S5–S7†). All synthesized MOFs are based on carboxylic acids, and so the charge transfer from the linker to the metal is a common phenomenon that results in broad peaks.^61–64^

Analyzing the Tauc plots by applying the formula (*αhv*)^2^ = *B*(*hv* − *E*_g_) led to the bandgap energy values (*E*_g_) of MOFs and composites. In the formula, *hv*, *α*, *B*, and *v* are the photo energy, absorption coefficient, proportionality constant, and the light frequency. Therefore, the calculated bandgap energies of CAU-1/Ce-BDC–NH_2_, CAU-1/Ce-UiO-66, CAU-1/Ce-MOF-808, CAU-1/Ce-BDC are 2.5, 2.75, 2.75, and 2.7 eV, respectively (Fig. 4b). The bandgap energies of CAU-1/Ce-BDC–NH_2_ containing –NH_2_ group in their structure were found to be lower than those of other heterostructures. The existence of less electronegative N 2p orbitals is the reason for their lower bandgap energies.^44,65^ To compare the band structure of CAU-1/Ce-BDC with bare MOFs, the UV-vis DRS of CAU-1 and Ce-BDC are displayed in Fig. 4d. CAU-1 and Ce-BDC showed *E*_g_ values of 2.6 eV and 2.8 eV, respectively.

**Fig. 5:**
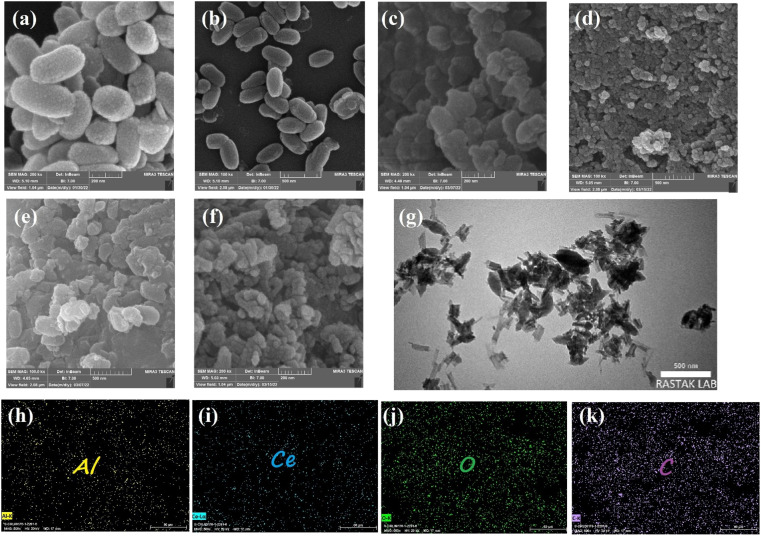
FE-SEM images of samples: (a, b) CAU-1, (c) CAU-1/UiO-66, (d) CAU-1/MOF-808, (e) CAU-1/Ce-BDC–NH_2_, and (f) CAU-1/Ce-BDC. (g) TEM images and (h–k) elemental mapping of the CAU-1/Ce-BDC heterostructure.

Scanning electron microscopy was employed to further study all samples' microstructure. As shown in Fig. 5a and b, CAU-1 has a rice kernel shape morphology, and exhibits uniform particle size and smooth surfaces.^41^ Other pristine MOFs, namely, Ce-BDC–NH_2_, Ce-BDC, Ce-MOF-808, and Ce-UiO-66 illustrated the amounts of intergrown nanoparticles (Fig. S8†).^55,66,67^ Combining Ce-MOFs with CAU-1 can enlarge the aggregate surface area, a key factor in active sites' provision. Fig. 5c–f demonstrates the SEM images of CAU-1/Ce-UiO-66, CAU-1/Ce-MOF-808, CAU-1/Ce-BDC–NH_2_, and CAU-1/Ce-BDC heterojunctions, which prove the successful synthesis of MOF-on-MOF structures. Some slight agglomerations observed in images can be attributed to dissimilar networks of MOFs, which inhibit achieving uniform structures. For a detailed exploration, the growth of Ce-on the surface of CAU-1, as two non-isostructural MOFs, was studied by transmission electron microscopy (TEM). According to Fig. 5g, CAU-1 is surrounded by Ce-BDC, as can be seen in dark gray. The lighter parts around it represent Ce-BDC. The synergic effect that appears after heterojunction formation is the reason for the high enhancement in properties.^68^

The elemental maps for CAU-1/Ce-BDC are presented in Fig. 5h–k. The surface of CAU-1/Ce-BDC is composed of carbon, oxygen, aluminium, and cerium. The simultaneous presence of Al and Ce that are related to CAU-1 and Ce-based MOFs, respectively, and the homogeneous distribution of elements, which was deduced from mapping images, proves the complete synthesis of heterojunctions.

### Photocatalytic Cr(vi) reduction activities

#### Photocatalytic Cr(vi) reduction

The photocatalytic activity toward reducing Cr(vi) was evaluated, which was performed in a reaction system using benzyl alcohol as the hole scavenger. The UV-vis spectra of Cr(vi) for all samples were recorded at different time intervals (every 15 min). It should be noted that bare CAU-1 did not show any photocatalytic activity under the reaction condition. Still, due to the large surface area of this framework (BET surface area is about 1816 m^2^ g^−1^ (Fig. S9†)), it can be utilized as an adsorbent along with other metal–organic frameworks. However, Ce(iv)-based MOFs were noticed because the use of Ce(iv) allows us to get MOFs isostructural to Zr homologs with remarkable stability and potential interest in the fields of redox and photocatalysis. Incorporating Ce-based MOFs onto the CAU-1 surface could lead to hybrid materials with the characteristics of both MOFs.

**Fig. 6:**
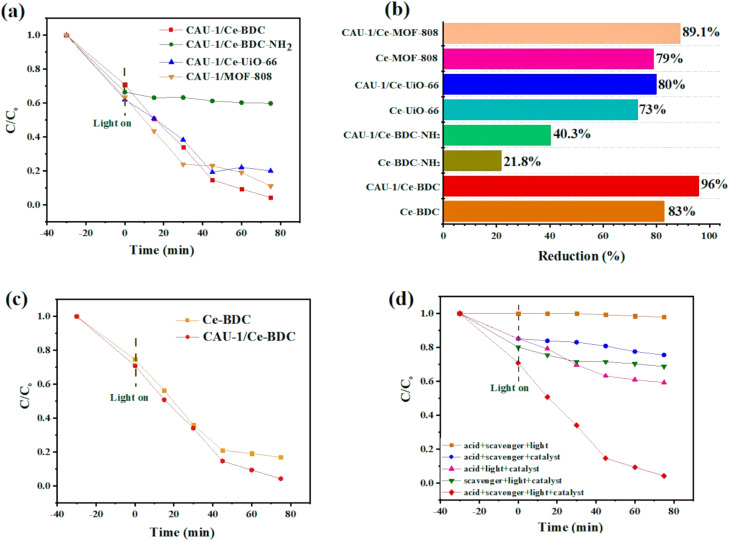
(a) Photocatalytic Cr(vi) reduction dynamics plots over CAU-1/Ce-UiO-66, CAU-1/Ce-MOF-808, CAU-1/Ce-BDC–NH_2_, and CAU-1/Ce-BDC, (b) reduction efficiency for all samples, (c) time-dependent plots for CAU-1/Ce-BDC and Ce-BDC, and (d) time-dependent plots under various conditions.

To confirm this hypothesis, CAU-1/Ce-UiO-66, CAU-1/Ce-MOF-808, CAU-1/Ce-BDC–NH_2_, and CAU-1/Ce-BDC constructed *via* a MOF-on-MOF synthesis strategy and the chromium reduction reaction was selected as a model experiment to testify to the effect of the new materials. Fig. 6a illustrates the photocatalytic activity of CAU-1/Ce-UiO-66, CAU-1/Ce-MOF-808, CAU-1/Ce-BDC–NH_2_, and CAU-1/Ce-BDC heterojunctions toward Cr(vi) reduction reactions. It was shown that the composition of CAU-1 with Ce-based MOFs can enhance the photocatalytic performance of cerium family MOFs; thus, these new MOF-on-MOF structures can reduce Cr(vi) to Cr(iii) (Fig. 6b). Cr(vi) reduction efficiency was calculated for CAU-1/Ce-UiO-66, CAU-1/Ce-MOF-808, CAU-1/Ce-BDC–NH_2_, and CAU-1/Ce-BDC to be 80%, 89.1%, 40.3%, and 96%, respectively within 75 min of reaction time. Despite the presence of amino groups in CAU-1/Ce-BDC–NH_2,_ the insufficient photocatalytic activity was attributed to this structure's slight BET surface area (BET surface area is about 154 m^2^ g^−1^ (Fig. S9†)).

Fig. 6c compares the photocatalytic performance and kinetic curves for Ce-BDC and CAU-1/Ce-BDC. This high chromium reduction efficiency could be attributed to the high absorption efficiency of CAU-1/Ce-BDC in the UV-vis region, its low bandgap, high surface area, and enhanced light harvesting. Cr(vi) photoreduction is a light-dependent reaction, but besides this, different parameters including pH, hole scavenger, catalyst/Cr(vi) dosage, and water source play vital roles. Acidic media facilitate absorbing negative HCrO_4_^−^ on the protonated surface of the catalyst, and the scavenger boosts the hole (h^+^) consuming *via* enhancing the separation efficiency of the photogenerated electrons and holes. The reaction did not progress considerably in the absence of either photocatalyst, hole scavenger, acid, or light irradiation (Fig. 6d). Therefore, this photoreduction reaction was conducted under controlled conditions.^69–71^

#### Influence of hole scavengers

**Fig. 7:**
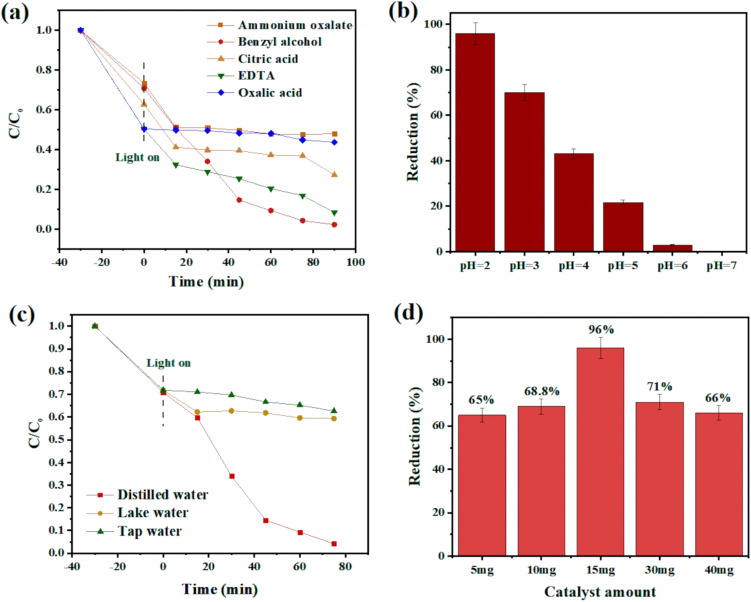
(a) Time-dependent plot of CAU-1/Ce-BDC in the presence of different hole scavengers, (b) chromium reduction efficiency at pH 2–7, (c) comparison of various water sources, and (d) study of the effect of catalyst dosage on the chromium photoreduction.

Hole scavengers are another critical factor in photocatalytic systems. After light irradiation, electron–hole pairs will be generated, and fast hole (h^+^) consumption can accelerate the reduction rate.^72^ To test the effect of consuming holes toward Cr(vi) removal, various hole scavengers such as oxalic acid, citric acid, benzyl alcohol, EDTA, and ammonium oxalate were chosen at a pH of 2.0. Among these candidates, benzyl alcohol was the most effective one, which could dramatically boost the reaction rate (Fig. 7a).

#### Influence of initial pH

Previous research has revealed that the photoreduction activity could be improved under low-pH conditions.^73,74^ Adjusting the solution's acidity by adding HCl makes the catalyst's surface protonated, simplifying HCrO_4_^−^ adsorption with strong electrostatic interactions. As shown in Fig. 7b, as the pH increases, the reaction rate decreases due to the conversion of HCrO_4_^−^ to more negative species such as CrO_4_^2−^ and Cr_2_O_7_^2−^ preventing the attraction of anionic chromate to the surface. This repulsion causes low catalytic efficiency. According to the results, pH 2 was chosen as the favourable pH for the subsequent reaction because of the maximum adsorption efficiency of the catalyst.^75,76^

#### Influence of water sources

The existence of organic pollutants and inorganic ions such as Na^+^, K^+^, Ca^2+^, Mg^2+^, Cl^−^, SO_4_^2−^, NO_3_^−^, and PO_4_^3−^ is inevitable in real nature. Therefore, the experiment was conducted using actual tap and lake water. As expected, the chromium removal percentage exposed a significant decrease compared to distilled water, and this may have been ascribed to the ions' interference during the photoreduction process^77^ (Fig. 7c).

#### Influence of photocatalyst dosages

As shown in Fig. 7d, the influences of photocatalyst dosages on Cr(vi) reduction were investigated by varying the dosages of CAU-1/Ce-BDC as 5 mg, 10 mg, 15 mg, 30 mg, and 40 mg. With the increase in CAU-1/Ce-BDC dosage up to 15 mg, the removal efficiency of Cr(vi) increased to 96% due to more active sites for photocatalytic reactions. When the dosage increased from 15 to 40 mg, the Cr(vi) reduction efficiency was not significantly improved. It is likely because the excessive particles might consequently hinder the light penetration.

### Reusability and stability of CAU-1/Ce-BDC

**Fig. 8:**
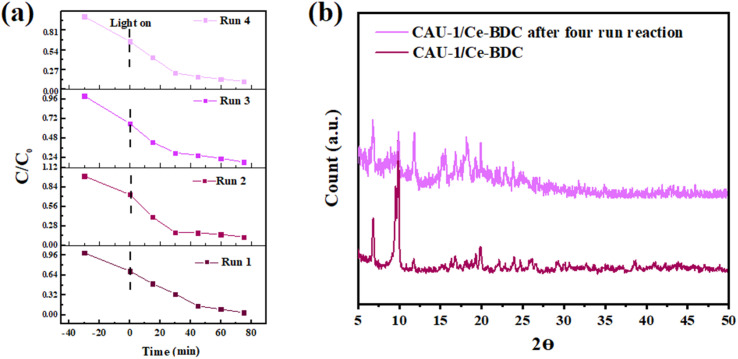
(a) Cycling test and (b) powder X-ray diffraction pattern of CAU-1/Ce-BDC after four-run reactions for chromium reduction.

From an economic point of view, having considerable chemical and structural stability is crucial for a promising photocatalyst. To check the stability and also reusability of CAU-1/Ce-BDC, four successive Cr(vi) reduction processes were conducted as recycling experiments (Fig. 8a). The catalyst was separated through centrifugation and utilized as the catalyst for the next run. No significant change was observed in Cr(vi) photoreduction efficiency. Additionally, the PXRD patterns of CAU-1/Ce-BDC before and after the reaction were similar without notable phase change in the structure (Fig. 8b). Thus, the excellent stability and reusability of the catalyst were confirmed after the reaction.

### The mechanism of photocatalytic Cr(vi) reduction

Based on the experimental results, CAU-1/Ce-BDC reached its best performance *via* optimizing the influential factors in photocatalytic efficiency. Eqn (1)–(7) summarize the reaction mechanism. The adsorption of Cr(vi) ions into the CAU-1/Ce-BDC is the first step in Cr(vi) photoreduction. Cr(vi) ions adsorb abundantly on the surface of CAU-1/Ce-BDC. The photo-excited catalytic process would begin under visible light illumination when the adsorption–desorption equilibrium was attained. At pH 2, where there is the greatest electrostatic interaction between the positively charged catalyst and the Cr(vi) anions present in the form of HCrO_4_^−^ and Cr_2_O_7_^2−^ ions, CAU-1/Ce-BDC demonstrates its maximum performance.^78,79^ These Cr(vi) ions can be reduced and changed into Cr(iii) using the electrons generated by the CAU-1/Ce-BDC photocatalyst, as shown in eqn (1)–(5):

1 CAU-1/Ce-BDC + *hv* → CAU-1/Ce-BDC (e^−^ + h^+^)

2 Cr_2_O_7_^2−^ + 14H^+^ + 6e^−^ → 2Cr^3+^ + 7H_2_O

3 HCrO_4_^−^ + 7H^+^ + 3e^−^ → Cr^3+^ + 4H_2_O

4 2H_2_O →O_2_ + 4H^+^ + 4e^−^

5 H_2_O + h^+^ → ˙OH + H^+^

6 ˙OH + hole scavenger → … → CO_2_ + H_2_O

7 h^+^ + hole scavenger → … → CO_2_ + H_2_O

The electron excitation from the valence band to the conduction band of CAU-1/Ce-BDC caused by light irradiation produces electron–hole pairs. The photogenerated holes are scavenged readily by the hole scavenger and prevent electron and hole recombination to retain the photoreduction of Cr(vi) in the aqueous solution (eqn (6) and (7)).^80^Fig. 11 illustrates the possible mechanism of photocatalytic Cr(vi) reduction reaction.

### Electrochemical measurements

**Fig. 9:**
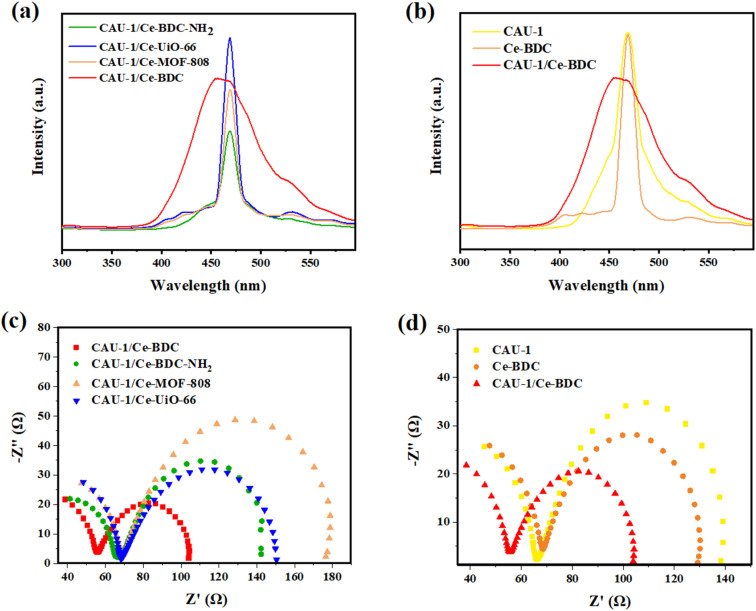
(a) PL spectra of CAU-1/Ce-BDC–NH_2_, CAU-1/Ce-BDC, CAU-1/Ce-MOF-808, and CAU-1/Ce-UiO-66 heterojunctions, (b) comprehensive PL spectra of CAU-1/Ce-BDC with CAU-1 and Ce-BDC, (c) EIS Nyquist impedance plots of CAU-1/Ce-BDC–NH_2_, CAU-1/Ce-BDC, CAU-1/Ce-MOF-808, and CAU-1/Ce-UiO-66 heterojunctions, and (d) comprehensive EIS Nyquist impedance plots of CAU-1/Ce-BDC with CAU-1 and Ce-BDC.

The photoluminescence (PL) and electrochemical impedance spectroscopy (EIS) measurements were applied to investigate the separation efficiency of photoinduced electron–hole pairs. Photoluminescence can provide valuable information about the recombination and capture of charge carriers in semiconductors. Fig. 9a shows the PL spectra of CAU-1/Ce-BDC, CAU-1/Ce-BDC–NH_2_, CAU-1/Ce-UiO-66, and CAU-1/Ce-MOF-808 in the range of 300–600 nm at the center of about 470 nm. As illustrated in Fig. 9a, it can be found that the PL intensity followed the order of CAU-1/Ce-UiO-66 > CAU-1/Ce-BDC > CAU-1/Ce-MOF-808 > CAU-1/Ce-BDC–NH_2._ The comparison of the photoluminescence of CAU-1, Ce-BDC, and CAU-1/Ce-BDC is displayed in Fig. 9b. Based on the results, the CAU-1/Ce-BDC heterojunction displayed a lower PL intensity than that of pristine CAU-1 and Ce-BDC. In this regard, the excited electron–hole in the CAU-1/Ce-BDC hybrid can migrate between CAU-1 and Ce-BDC. As the PL intensity decreases, the structure's potential in electron–hole pair separation increases, which results in slower recombination of photoinduced charge carriers. This phenomenon can positively affect the photocatalysis procedure.

The electrochemical behavior of all synthesized heterojunctions and their components toward charge separation resistance was studied by electrochemical impedance spectroscopy.^81^Fig. 9c demonstrates the Nyquist plots of CAU-1/Ce-UiO-66, CAU-1/Ce-MOF-808, CAU-1/Ce-BDC–NH_2_, and CAU-1/Ce-BDC MOF-on-MOF structures. According to the arc area of Nyquist plots in Fig. 9c, the decrease in the radius of circles results from less resistance in charge transfer. Among the heterojunctions, CAU-1/Ce-BDC displayed the smallest semicircle arc, indicating facile charge separation. Despite its high photoluminescence emission, the excellence of CAU-1/Ce-BDC performance in photocatalysis might be attributed to the lowest electron transfer resistance, even compared to pristine CAU-1 and Ce-BDC.^42^ Moreover, Fig. 9d compares two bare CAU-1 and Ce-BDC MOFs with the CAU-1/Ce-BDC heterojunction. The results indicated that the interfacial resistance of CAU-1/Ce-BDC is lower and has more efficient electron–hole pair separation and charge transfer.

**Fig. 10:**
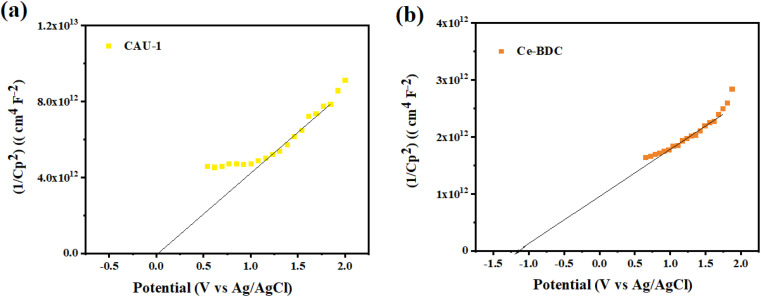
Mott–Schottky plots of (a) CAU-1 and (b) Ce-BDC.

To determine the type of semiconductor and calculate the position of the conduction band (CB) and valence band (VB), the Mott–Schottky (MS) test was carried out (Fig. 10). The results illustrate that the tangent slopes of CAU-1 and Ce-BDC are positive, indicating that they belong to an n-type semiconductor. By fitting the linear region of the Mott–Schottky plots, the flat band potential (*E*_FB_) can be determined at the *y*-intercept. Ce-BDC demonstrates the most negative *E*_FB_ value around −1.2 V *vs.* Ag/AgCl, which is in line with that of mesoporous Ce-BDC (Fig. 10b). Similarly, CAU-1 exhibits an *E*_FB_ value of 0 V *vs.* Ag/AgCl (Fig. 10a). Commonly, the flat band potential of the n-type semiconductor is about 0.10 eV higher than that of the conduction band (*E*_CB_); therefore, the conduction band (LUMO) of CAU-1 and Ce-BDC was equal to 0.1 eV and −1.1 eV *versus* Ag/AgCl. However, according to the Kubelka–Munk plot of energy (eV) *vs.* (*αhv*), the band gap values of CAU-1 and Ce-BDC were estimated at 2.7 and 2.8 eV, respectively. *Via* analyzing the UV-vis diffuse reflectance spectrum to obtain the bandgap data of the sample, it can be concluded that the valence band potentials (*E*_VB_) are 2.89 and 1.79 eV for CAU-1 and Ce-BDC electrodes, respectively.

**Fig. 11:**
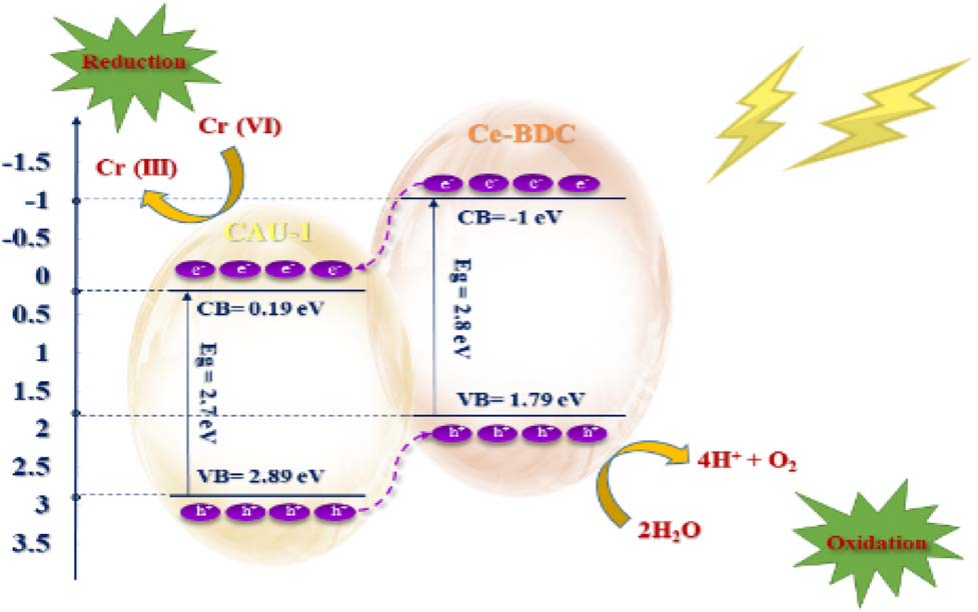
Possible mechanism of Cr(vi) reduction over CAU-1/Ce-BDC.

## Conclusion

In summary, four Ce-based MOFs were chosen to be synthesized in the presence of CAU-1, as a dissimilar MOF. Eventually, we claimed proper synthesis of CAU-1/Ce-BDC–NH_2_, CAU-1/Ce-UiO-66, CAU-1/Ce-MOF-808, and CAU-1/Ce-BDC, and various characterization techniques proved great enhancement in their structural properties. The photocatalytic performance of structures in Cr(vi) reduction reactions was explored as a proof of concept. Based on the results, the photocatalytic reaction progressed over 96% for the optimum structure, CAU-1/Ce-BDC, within 75 minutes. Moreover, its chemical and structural stability did not change significantly after four runs of reaction. Accordingly, we introduced four novel heterojunctions based on cerium, and the optimum structure exhibited excellent photocatalytic efficiency.

## Conflicts of interest

There are no conflicts to declare.

## Supplementary Material

RA-012-D2RA06034E-s001
