# Supplementary material for: Exploring novel heterojunctions based on the cerium metal–organic framework family and CAU-1, as dissimilar structures, for the sake of photocatalytic activity enhancement
Source: RSC Adv. 2022 Nov 10;12(50):32237–48. doi: 10.1039/d2ra06034e (PMC9647877; doi:10.1039/d2ra06034e)
Supplement: RA-012-D2RA06034E-s001 [file RA-012-D2RA06034E-s001.pdf]

Electronic Supplementary Material (ESI) for RSC Advances.
This journal is © The Royal Society of Chemistry 2022

Exploring novel heterojunctions based on cerium metal-organic frameworks' family and CAU-1, as dissimilar structures, for the sake of photocatalytic activity enhancement

Moein Darabi Goudarzi, Negin Khosroshahi, Vahid Safarifard*

Department of Chemistry, Iran University of Science and Technology, Tehran 16846-13114, Iran, E-mail: vsafarifard@iust.ac.ir

1. Preparation methods

1.1. Preparation of Ce-BDC-NH_2

As reported in the literature [1], CAN (1.5 mmol, 822.34 mg) and the linker BDC-NH_2 (6 mmol, 1.086 g) were suspended in the mixture of DMF (18 mL) and methanol (2 mL). After 30 min under magnetic stir, the resulting liquid was moved to a high-pressure Teflon-lined stainless-steel autoclave and kept in the oven for 72 h at 150 °C. Then, to remove unreacted BDC-NH_2, the brown precipitate was washed with DMF and methanol several times and kept in a vacuum oven at 100 °C for 12 h to reach Ce-BDC-NH_2 powder.

1.2. Preparation of Ce-UiO-66

Ce-UiO-66 was synthesized based on the previously reported method. First, CAN (0.533 M, 1.753 g) and H_2BDC (3.2 mmol, 531 mg) were dissolved in distilled water (6 mL) and DMF (12 mL), respectively. The aqueous solution was poured into the beaker containing the linker solution. The resulting mixture was placed in a preheated oil bath for 15 min at 100 °C. The white powder product was reached after washing and centrifuging several times with DMF and acetone. The solid powder was also dried in a vacuum oven at 100 °C for 12 h.

1.3. Preparation of Ce-MOF-808

To synthesize Ce-MOF-808, CAN (6 mL, 0.578 M) was added to another beaker that includes the solution of H_3BTC (0.106 mmol, 0.224 g) in DMF (12 mL). Next, 2.57 mL of formic acid was added to the mixture and transferred to a vial. It was heated at 120°C for 20 min. After centrifuging

and washing the precipitation several times with DMF and acetone, the result of white powder was obtained. The final product was activated in a vacuum oven at 100 °C for 12 h (CCDC code: 1509776).

1.4. Preparation of Ce-BDC

Ce-BDC was prepared by CAN and H_2BDC through the hydrothermal route. CAN (1 g) and H_2BDC (0.6 g) were dissolved in DMF (15 mL) and reacted in an autoclave for 12 h at 150 °C. The obtained solution was washed and centrifuged three times with DMF to reach white powder and dried in a vacuum oven for 12 h at 100 °C (CCDC code: 912350) [2].

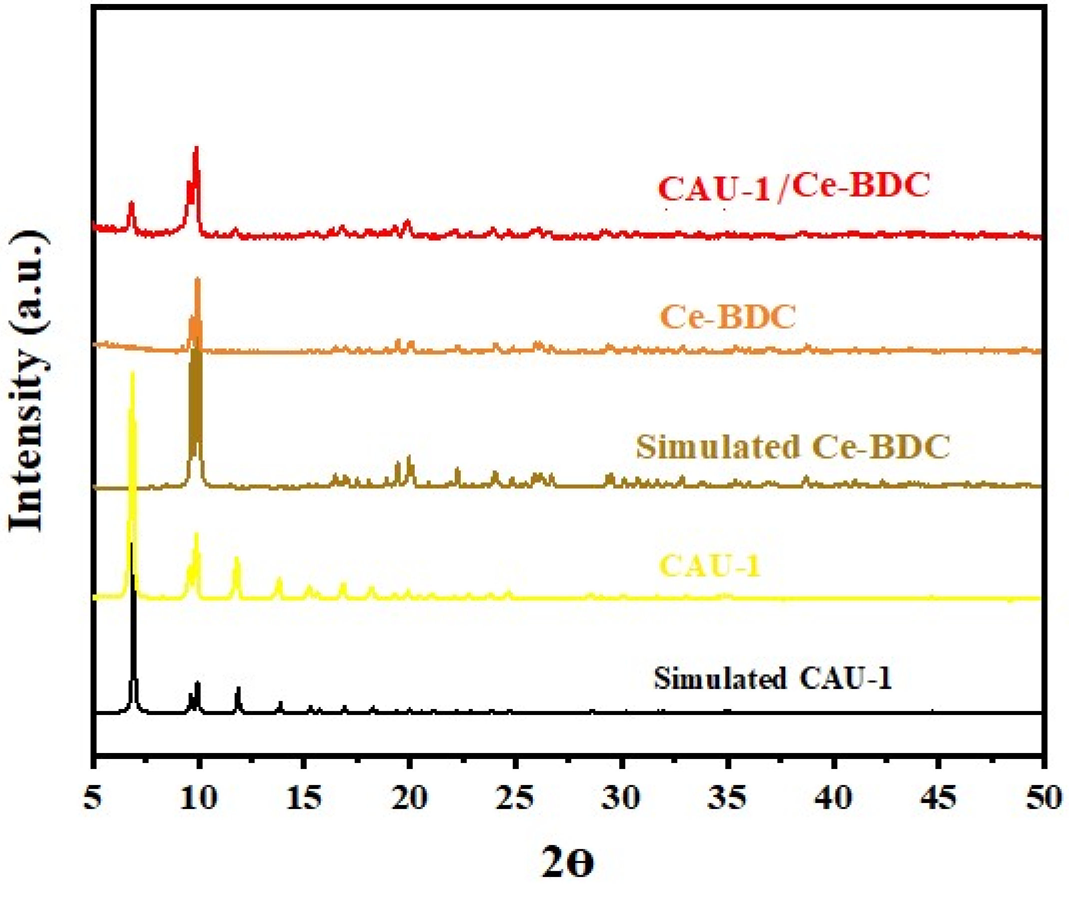

Figure S 1. Powder XRD pattern of Ce-BDC, CAU-1, and CAU-1/Ce-BDC.

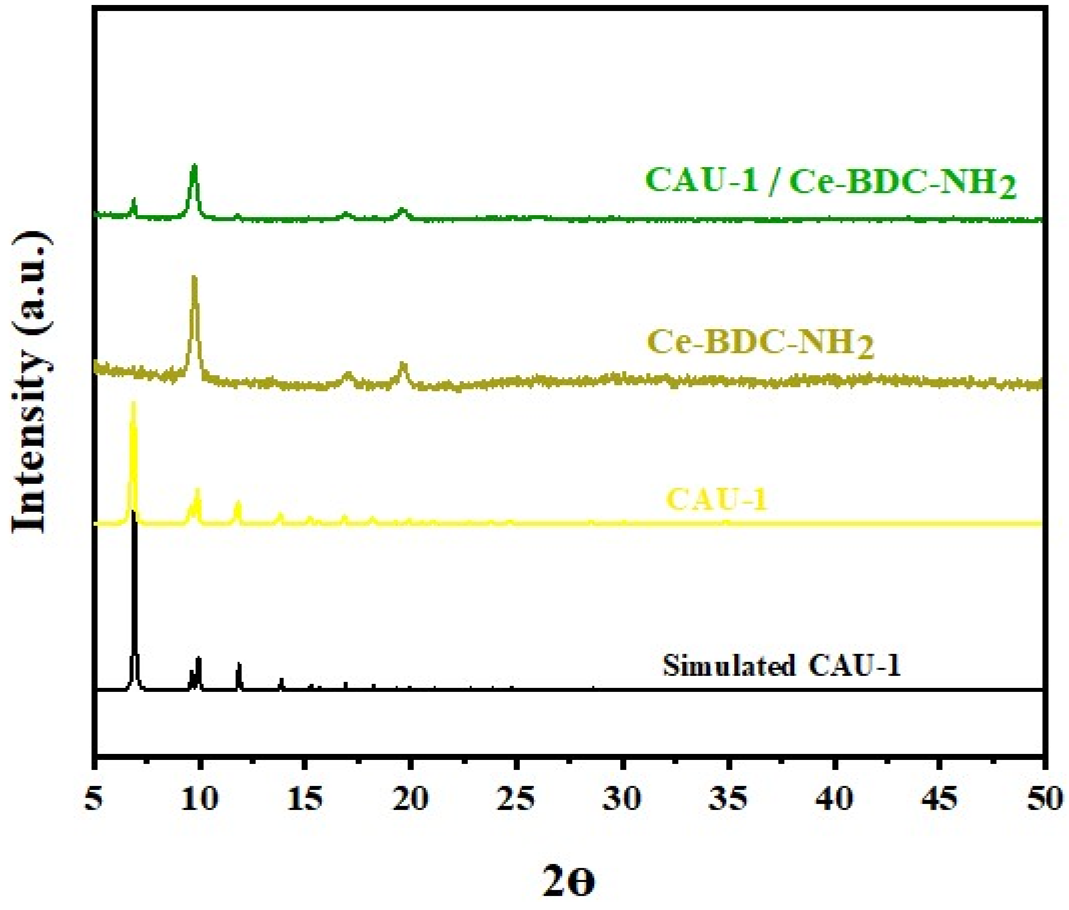

Figure S 2. Powder XRD pattern of Ce-BDC-NH_2, CAU-1, and CAU-1/Ce-BDC-NH_2.

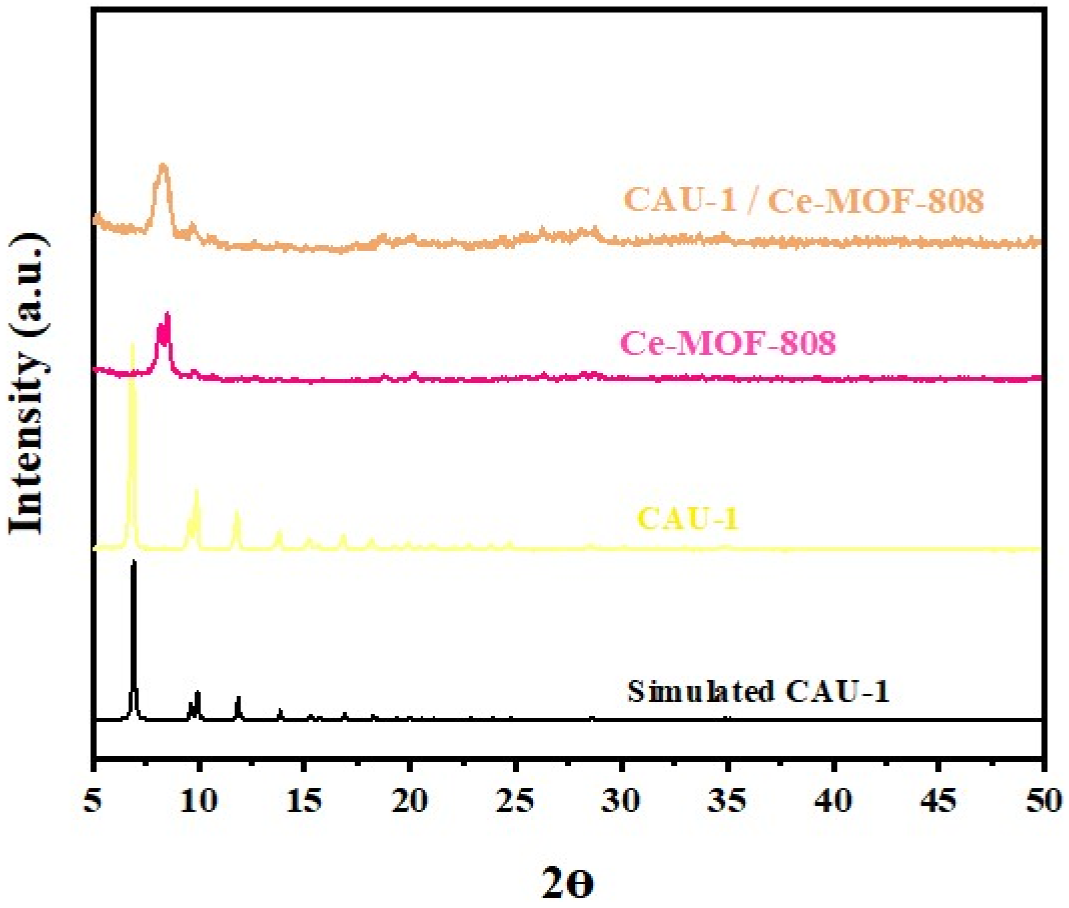

Figure S 3. Powder XRD pattern of Ce-MOF-808, CAU-1, and CAU-1/Ce-MOF-808.

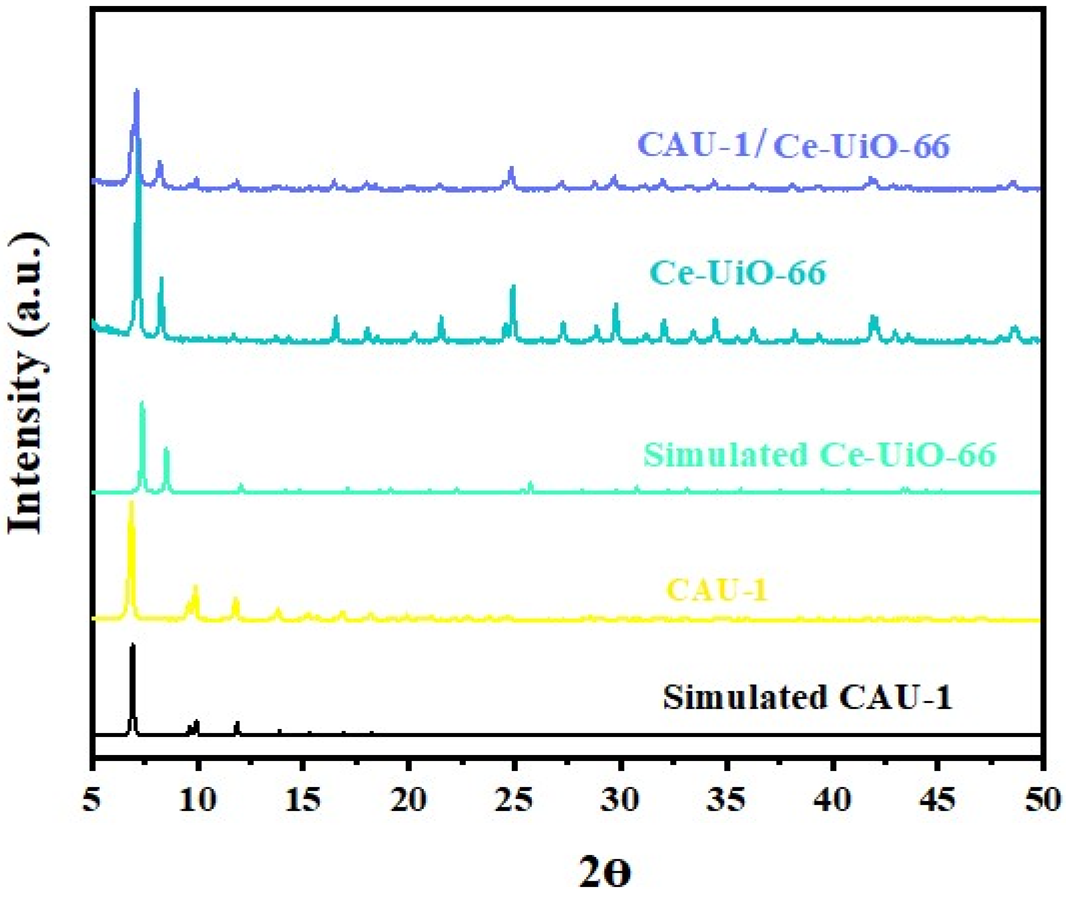

Figure S 4. Powder XRD pattern of Ce-UiO-66, CAU-1 and CAU-1/Ce-UiO-66.

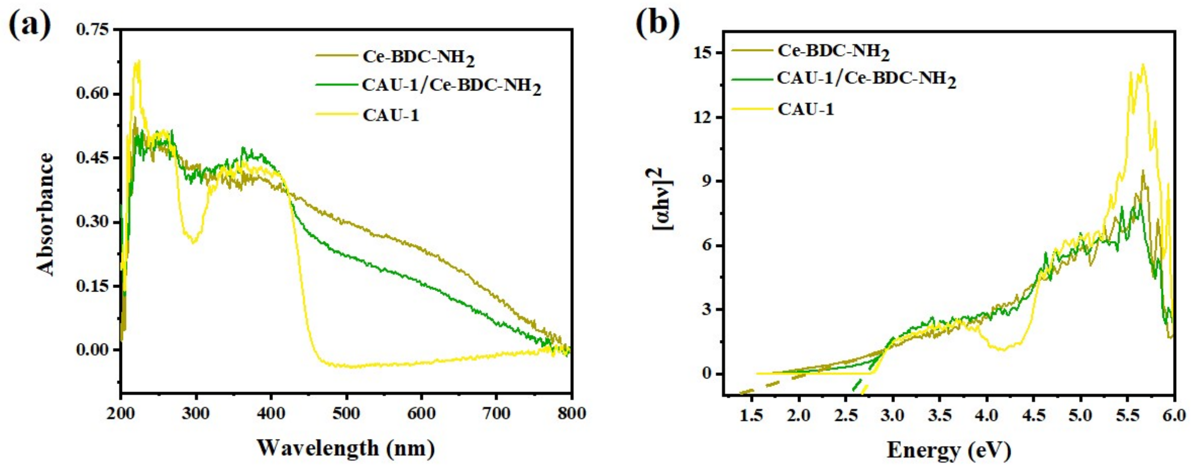

Figure S 5. (a) UV-Vis DRS spectra, (b) corresponding Tauc plots of $(\alpha h\nu)^2$ versus hν of Ce-BDC-NH_2, CAU-1, and CAU-1/Ce-BDC-NH_2.

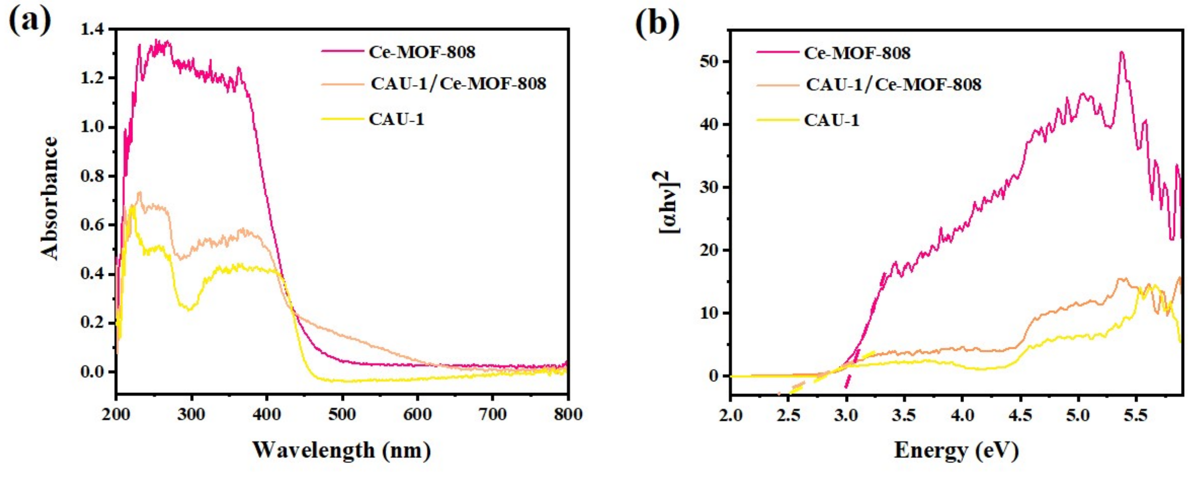

Figure S 6. (a) UV-Vis DRS spectra, (b) corresponding Tauc plots of $(\alpha h\nu)^2$ versus hν of Ce-MOF-808, CAU-1, and CAU-1/Ce-MOF-808.

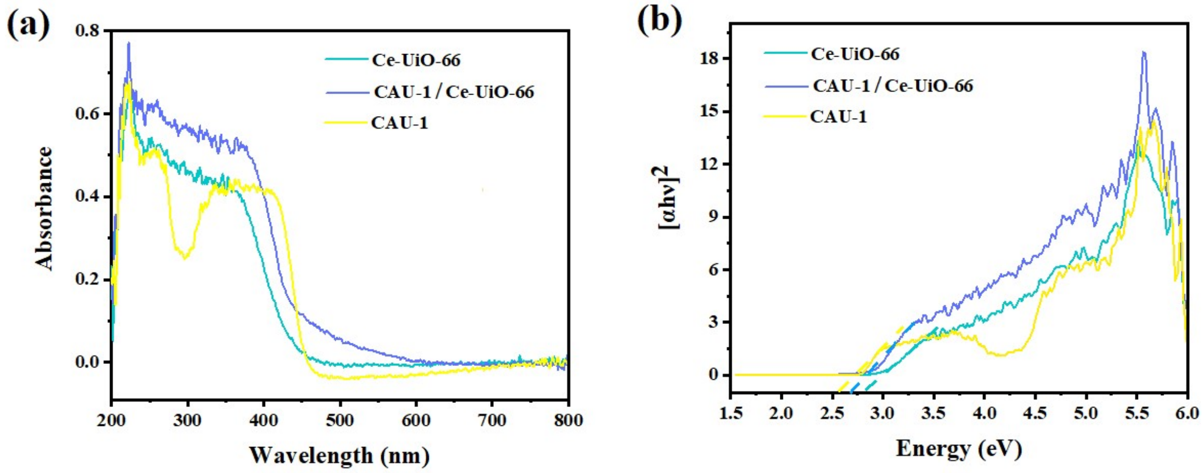

Figure S 7. (a) UV-Vis DRS spectra, (b) corresponding Tauc plots of $(\alpha h\nu)^2$ versus hν of Ce-UiO-66, CAU-1, and CAU-1/Ce-UiO-66.

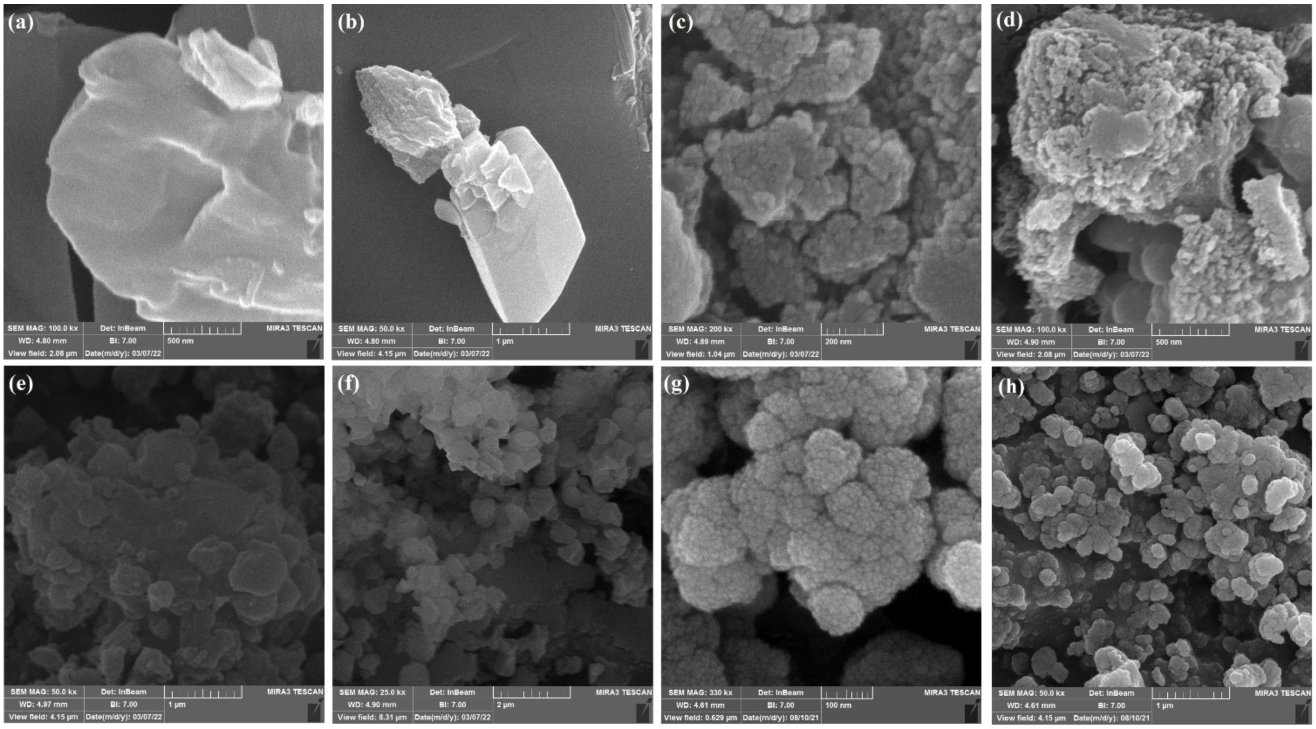

Figure S 8. (a, b) FE-SEM images of Ce-BDC, (c, d) Ce-BDC-NH_2, (e, f) Ce-UiO-66, and (g, h) Ce-MOF-808 in different scales.

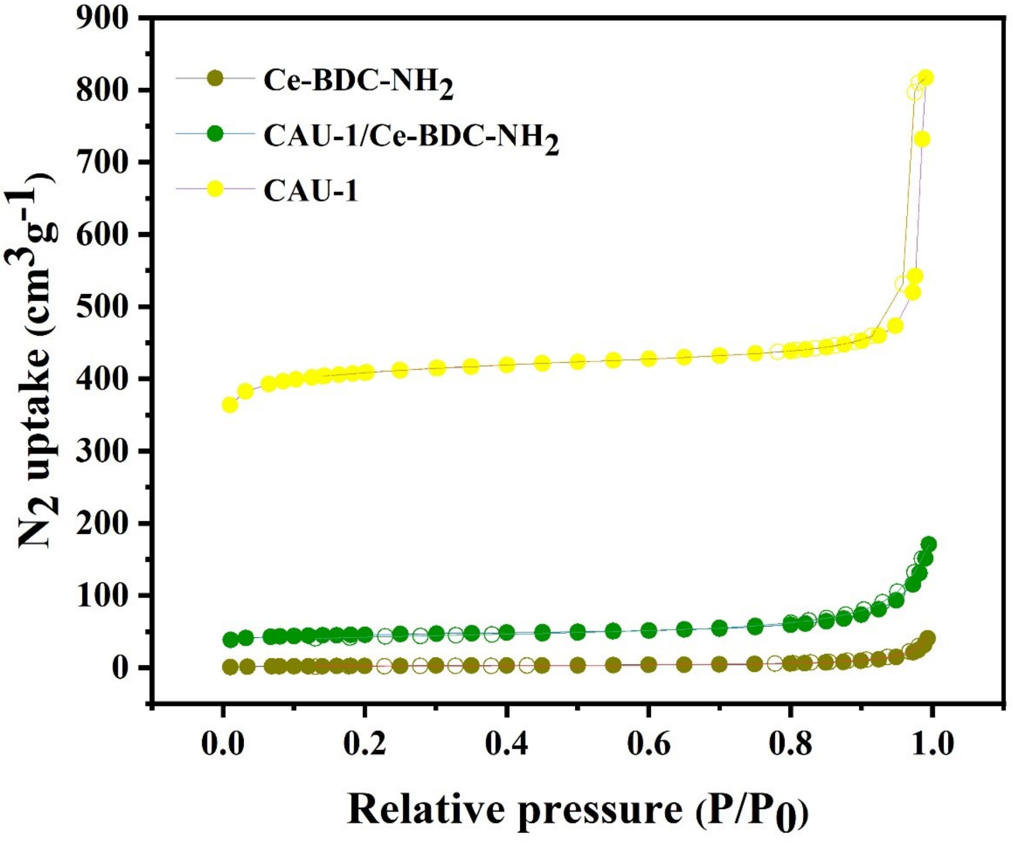

Figure S 9. N_2 adsorption/desorption isotherms of CAU-1, Ce-BDC-NH_2, and CAU-1/Ce-BDC-NH_2.

References

1. Payra, S. and S. Roy, *From Trash to Treasure: Probing Cycloaddition and Photocatalytic Reduction of CO2 over Cerium-Based Metal–Organic Frameworks.* The Journal of Physical Chemistry C, 2021. **125**(16): p. 8497-8507.
2. He, J., et al., *The structural design and valence state control of cerium-based metal-organic frameworks for their highly efficient phosphate removal.* Journal of Cleaner Production, 2021. **321**: p. 128778.
